# Synthesis of a water-soluble 2,2′-biphen[4]arene and its efficient complexation and sensitive fluorescence enhancement towards palmatine and berberine

**DOI:** 10.3762/bjoc.14.198

**Published:** 2018-08-27

**Authors:** Xiayang Huang, Xinghua Zhang, Tianxin Qian, Junwei Ma, Lei Cui, Chunju Li

**Affiliations:** 1 School of Chemical and Environmental Engineering, Shanghai Institute of Technology, 100 Hai-Quan Road, Shanghai 201418, P. R. China; 2 Department of Chemistry, Center for Supramolecular Chemistry and Catalysis, Shanghai University, Shanghai 200444, P. R. China

**Keywords:** berberine, biphenarenes, host–guest complexes, molecular recognition, palmatine

## Abstract

A water-soluble 2,2′-biphen[4]arene (2,2’-CBP4) containing eight carboxylato moieties was synthesized and characterized. Its complexation behavior towards two alkaloids, palmatine (**P**) and berberine (**B**), was investigated by means of fluorescence and ^1^H NMR spectroscopy in aqueous phosphate buffer solution (pH 7.4). In the presence of 2,2’-CBP4, ^1^H NMR signals of **P** and **B** displayed very large upfield shifts, indicating the formation of inclusion complexes with strong binding affinities. Fluorescence titration experiments showed that **P** and **B** exhibited dramatic fluorescence enhancement of more than 600 times upon complexation with 2,2’-CBP4. Particularly, the fluorescence intensity is strong enough to be readily distinguished by the naked eye. Although the two guests have similar structures, the association constant of **B** with 2,2’-CBP4 (*K*_a_ = (2.29 ± 0.27) × 10^6^ M^−1^) is 3.9 times larger than that of **P** (*K*_a_ = (5.87 ± 0.24) × 10^5^ M^−1^).

## Introduction

Host–guest chemistry in water is significantly important due to its extensive applications in biology, medicine, and environment. Cyclodextrins [1–4], cucurbiturils [5–11], and calixarenes [12–20] have been widely used in aqueous supramolecular chemistry. In the past ten years, the chemistry of pillar[*n*]arenes has developed very quickly because of their specific structures and interesting host–guest properties [21–32]. Water-soluble pillar[*n*]arene derivatives, especially those containing carboxylato moieties, showed low cell toxicity and good biocompatibility, and have been applied in biomedical applications such as bioimaging and self-assembled drug delivery systems [33–40]. For example, our group demonstrated a direct host–guest complexation-based drug delivery system for oxaliplatin by carboxylatopillar[6]arene [36]. The encapsulation could not only improve the drug‘s stability in the blood stream, but also be effectively dis-assembled in the acidic tumor environment, and thus improve the anticancer activity of oxaliplatin in vivo.

In 2015, we introduced a new class of macrocyclic arenes, 4,4’-biphen[*n*]arenes (*n* = 3,4) with 4,4’-biphenol or 4,4’-biphenol ether monomers linked by –CH_2_– bridges [41], which have received much attention due to their convenient synthesis and modification method, novel topological structures and excellent cavity host–guest properties [41–46]. In 2017, another type of biphen[*n*]arenes with 2,2’-disubstituted biphenyl units, 2,2’-biphen[*n*]arenes (*n* = 4–8), have been designed and synthesized [47].

**Scheme 1:**
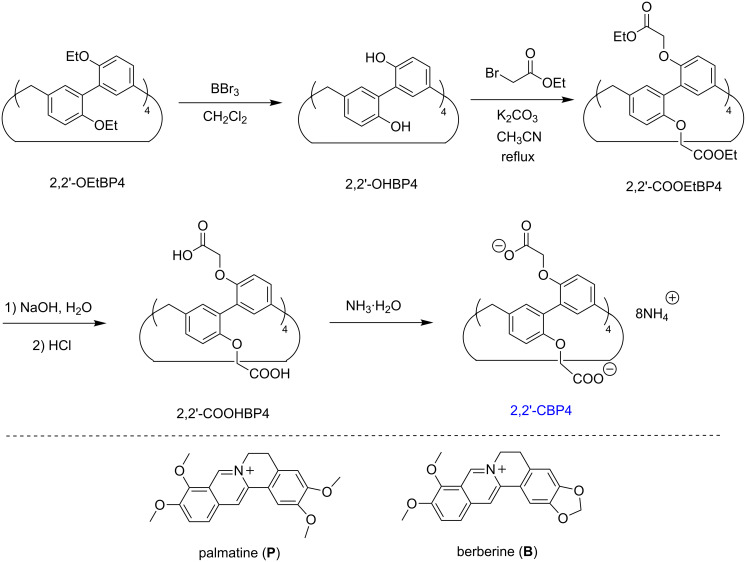
Synthesis of 2,2’-CBP4 and the chemical structures of **P** and **B**.

To date, the complexation of biological and pharmaceutical molecules by biphen[*n*]arenes in water have not been reported. In this work, we wish to report the synthesis of the first water-soluble 2,2’-biphen[4]arene bearing multiple carboxylato moieties, 2,2’-CBP4 (Scheme 1), and its binding behavior and fluorescent spectrum characteristic towards two alkaoilds, palmatine (**P**) and berberine (**B**), in water solution. In particular, the fluorescence intensities of the two guests have been considerably enhanced after complexation. As a member of isoquinoline alkaloids‘ family, **P** and **B** can produce singlet oxygen (^1^O_2_) and oxide biological substrates under light, and thereby have applications in photodynamic therapy (PDT) [48–50]. However, their low quantum yields limit such applications, which could be potentially improved or restored by the present encapsulation-induced fluorescence enhancement.

## Results and Discussion

### Synthesis

Scheme 1 shows the synthetic route of 2,2’-CBP4 [51], which is very similar with the procedure of water-soluble 4,4’-biphenarene [46]. Perhydroxylated 2,2’-biphen[4]arene, (2,2’-OHBP4) with hydroxy reaction sites was quantitatively prepared by the deprotection of 2,2’-OEtBP4 using excess BBr_3_. The nucleophilic substitution reaction of 2,2’-OHBP4 and ethyl bromoacetate, K_2_CO_3_ as the base, afforded 2,2’-COOEtBP4 in 88% yield. The hydrolysis of 2,2’-COOEtBP4 in NaOH solution and then acidification with HCl yielded 2,2’-COOHBP4 in a high yield of 87%. Water soluble 2,2’-CBP4 was quantitavely prepared by the acid-base reaction of 2,2’-COOHBP4 and aqueous ammonia solution. The total yield is up to 77%. As expected, 2,2’-CBP4 has a very good solubility (≥10 mM) in water.

### ^1^H NMR spectra

**Figure 1:**
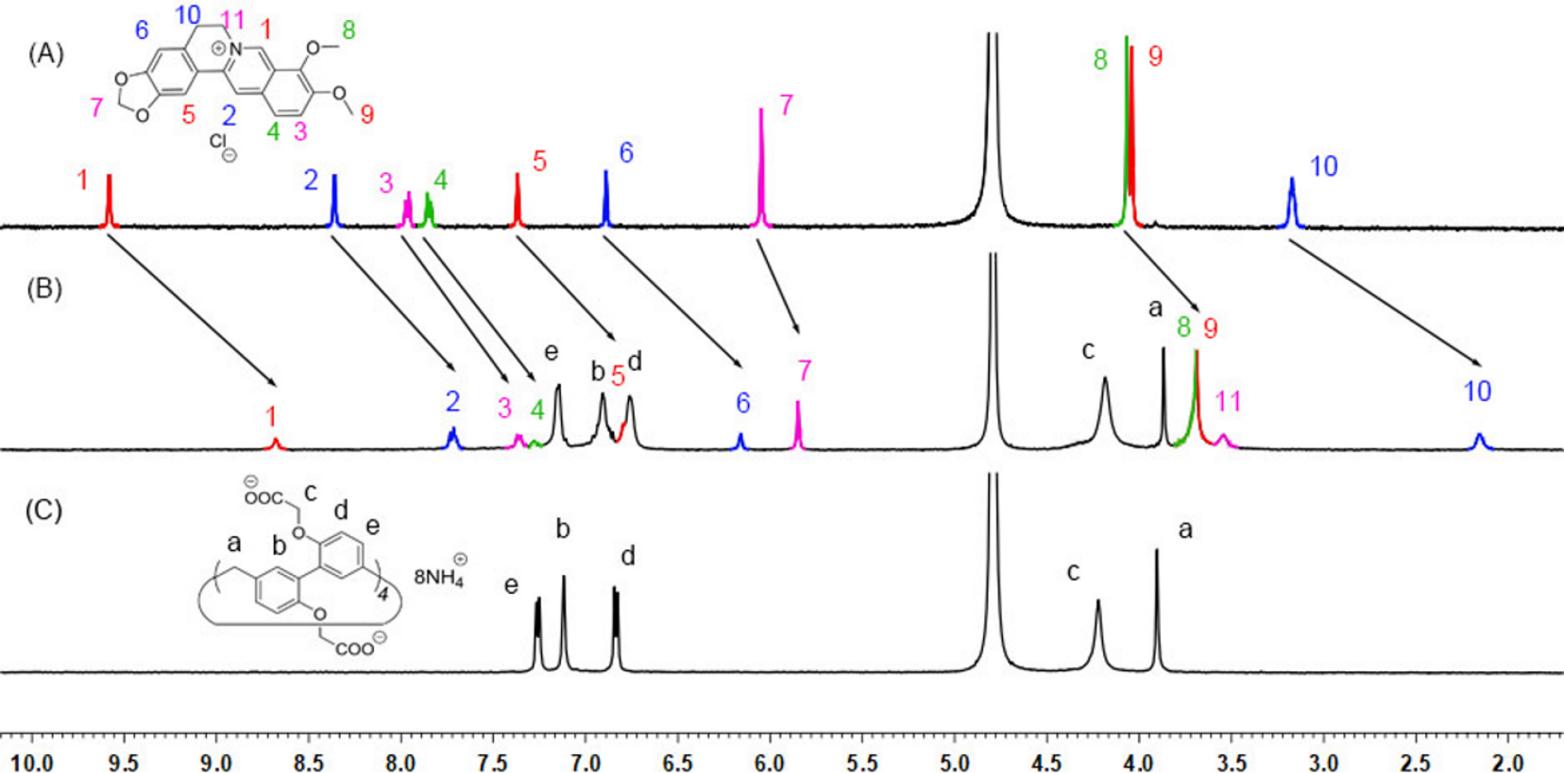
^1^H NMR spectra (500 MHz, 293 K) of (A) **B** (2.0 mM), (B) **B** (2.0 mM) + 2,2’-CBP4 (2.0 mM) and (C) 2,2’-CBP4 (2.0 mM) in deuterated phosphate buffer (pD 7.4).

^1^H NMR experiments of **P** and **B** with 2,2’-CBP4 in deuterated phosphate buffer (pD 7.4) were carried out to examine the host–guest complexation (Figure 1 and Figure S9 in Supporting Information File 1). From Figure 1, upon addition of the host, all the peaks of alkaloid **B** displayed upfield shifts and broadening compared with the free guest. Especially, the chemical shifts for the middle protons, H1–6_,_ and H10–11, are larger than those for the ending H7–9. These results indicate that berberine was engulfed by the cavity of 2,2’-CBP4 to form a pseudorotaxane-type inclusion complex. Similar complexation-induced NMR changes were observed for the host–guest mixture of **P** and 2,2’-CBP4 (Supporting Information File 1, Figure S9), suggesting a similar binding mode of an inclusion complex.

The host–guest encapsulation was then confirmed by 2D NOESY experiments, as shown in Figures S10 and S11, Supporting Information File 1. For example, in the 2D NOESY spectra of host–guest mixture of 2,2’-CBP4 and **B**, NOE correlations were clearly observed between the middle protons H1 and H10 of **B** with the methylene H_c_ of 2,2’-CBP4, and between the aromatic protons (H_b_) of 2,2’-CBP4 and H2 of **B** (Supporting Information File 1, Figure S11).

**Figure 2:**
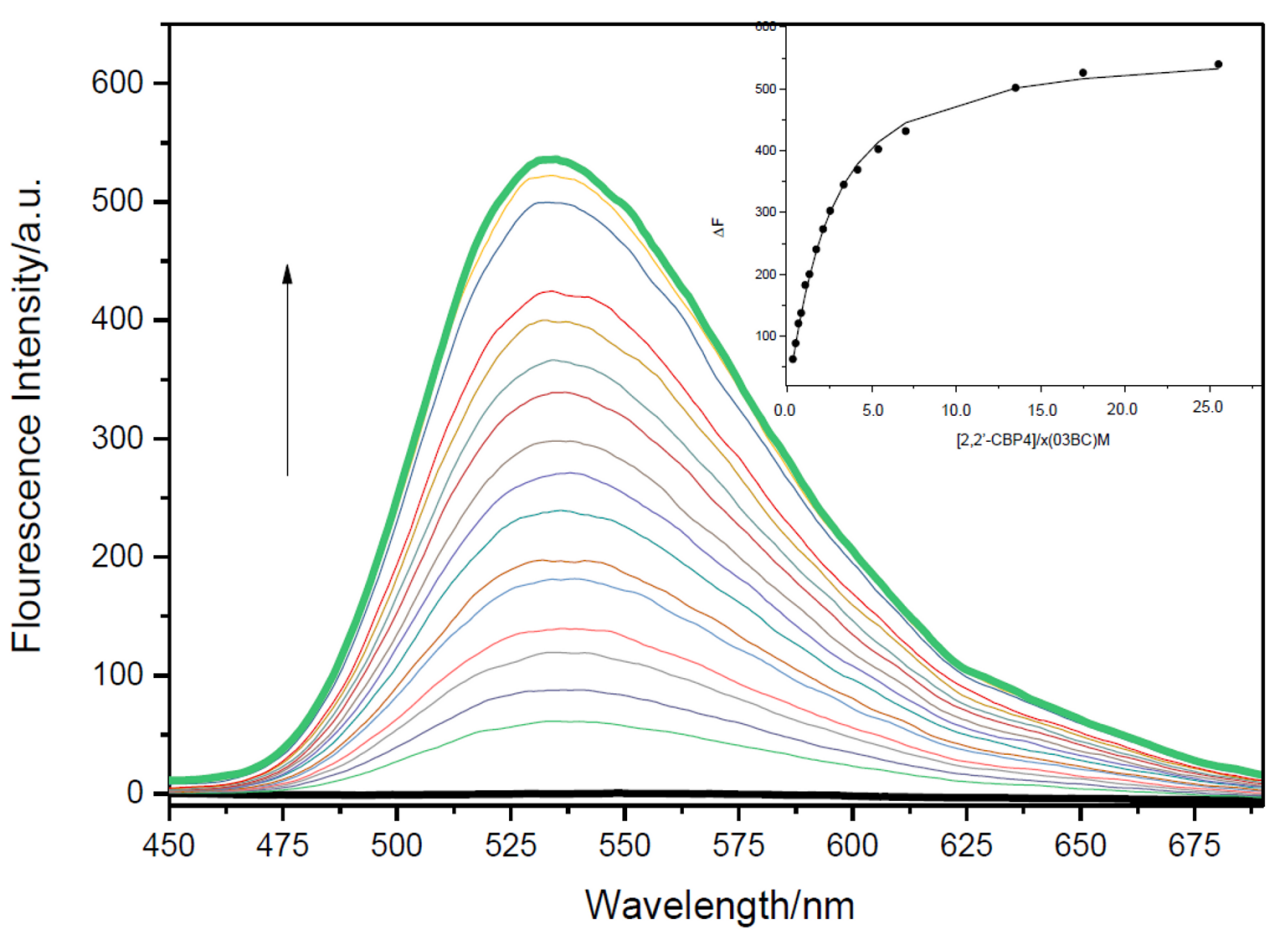
Fluorescence spectra of **P** in the absence and presence of 2,2’-CBP4 in aqueous phosphate buffer solution at pH 7.4 at 298 K. The excitation wavelength is at 352.0 nm. Inset: the nonlinear least-squares analysis to calculate the association constant using the fluorescence emission at 530 nm.

**Figure 3:**
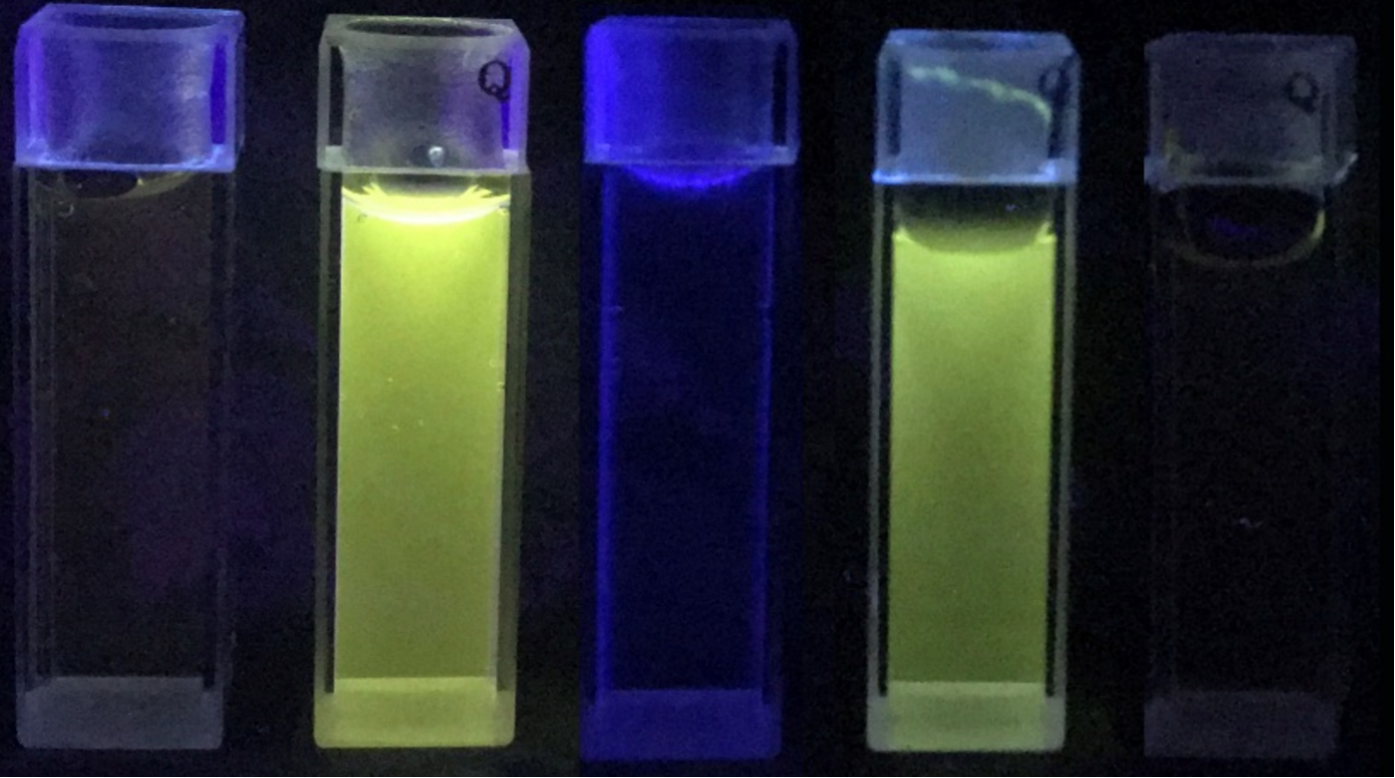
Visible emission observed from samples of **P** and **B** in the absence and presence of 2,2’-CBP4 under a UV lamp (365 nm). Left to right: **P**, **P** + 2,2’-CBP4, 2,2’-CBP4, **B** + 2,2’-CBP4 and **B**.

To examine the fluorecence behavior and to quantitatively assess the complexation of the two alkaloids and 2,2’-CBP4, spectral titrations of **P**/**B** and 2,2’-CBP4 were performed in the phosphate buffer solution of pH 7.4 at 298 K. As can be seen from Figure 2 and Supporting Information File 1, Figure S10, compounds **P** and **B** alone only displayed fairly feeble fluorescence emission. Upon addition of 2,2’-CBP4, the fluorescence intensity was remarkably improved more than 600 times (Figure 2 and Supporting Information File 1, Figure S10). This was due to the effect of lowering polar microenvironment when **P** or **B** was included by 2,2’-CBP4; the guest emits stronger fluorescence in a more hydrophobic microenvironment [48]. Combined with NMR results, we can unambiguously conclude the alkaloid molecules must insert into the hydrophobic cavity of 2,2’-CBP4 to form inclusion complexes. Interestingly, the emission intensities can be easily identified by the naked eye under UV light of 365 nm. As can be seen from Figure 3, **P**, **B** and 2,2’-CBP4 alone are almost nonfluorescent; the host–guest mixture shows very strong yellow fluorescence.

Through analyzing the sequential changes about fluorescence intensity (Δ*F*) of guest that occurred with changes in host concentration, the association constants (*K*_a_) could be calculated. The complexation stoichiometry for each binding event was determined to be 1:1 by Job plot analysis (Supporting Information File 1, Figures S13 and S14). The nonlinear least-squares curve-fitting method was used to analysis. For each host–guest pair, an excellent fit with an R value larger than 0.99 was obtained. It was found that 2,2’-CBP4 formed stable complexes with the two positively charged alkaloids, giving *K*_a_ values of (5.87 ± 0.24) × 10^5^ M^−1^ and (2.29 ± 0.27) × 10^6^ M^−1^ for **P** and **B**, respectively. π···π interactions, hydrophobic interactions and electrostatic attractions should play important roles in the association process. Although having similar structures, these two guests gave very different association constants. The substitution of 1,3-dioxole for two methoxy groups in **P**, affording **B**, considerably increases the *K*_a_ value of 3.9 times (Table 1). One possible reason is that the size of **B** with 1,3-dioxole, smaller than that for **P** with two methoxy groups, matches better with the cavity of 2,2’-CBP4.

**Table 1:** Association constants (*K*_a_/M^−1^) for 1:1 intermolecular complexation of **P** and **B** with 2,2’-CBP4 in phosphate buffer solution (pH 7.4) at 298 K.

host	guest	*K*_a_ [M^−1^]	Ex [nm]	Em [nm]

2,2’-CBP4	**P**	(5.87 ± 0.24) × 10^5^	352	533
2,2’-CBP4	**B**	(2.29 ± 0.27) × 10^6^	352	530

## Conclusion

In summary, we have synthesized a water-soluble 2,2’-biphen[4]arene, 2,2’-CBP4, for the first time and studied its complexation towards two alkaloid guests, **P** and **B**. ^1^H NMR and fluorescence results indicate the formation of inclusion complexes with strong stability. The association constants are in the magnitude of 10^5^–10^6^ M^−1^. Upon complexation with 2,2’-CBP4, both alkaloid guests exhibit a significant fluorescence intensity enhancement and the intensity is strong enough to be distinguished by the naked eye. The easy accessibility, good water-solubility and nice binding properties make 2,2’-CBP4 be applicable in the biomedical field, for example, chemical sensors, drug delivery, supramolecular amphiphiles, etc.

## Experimental

2,2’-OEtBP4 was synthesized according to our previously reported method [47]. **P** and **B** were purchased from Shanghai Aladdin Bio-Chem Technology Co.,LTD. ^1^H NMR and ^13^C NMR spectra were recorded on a Bruker AV500 instrument. The fluorescence emission spectra were determined with a SHIMADZU RF5301 spectrometer. Deuterated phosphate buffer solutions (20 mM) of pD 7.4 for ^1^H NMR experiments were prepared by mixing K_2_DPO_4_ deuterium oxide solution (20 mM) and KD_2_PO_4_ deuterium oxide solution (20 mM) according to the calculated volume ratios. The pH/pD values of the buffer solutions were verified on a pH-meter calibrated with two standard buffer solutions.

## Supporting Information

File 1 Experimental details and the ^1^H and ^13^C NMR spectra of 2,2’-biphen[4]arene derivatives, additional ^1^H NMR spectra of host–guest mixture, job plots, and the determination of the association constants.
