# Supplementary material for: Synthesis of a water-soluble 2,2′-biphen[4]arene and its efficient complexation and sensitive fluorescence enhancement towards palmatine and berberine
Source: Beilstein J Org Chem. 2018 Aug 27;14:2236–41. doi: 10.3762/bjoc.14.198 (PMC6122385; doi:10.3762/bjoc.14.198)
Supplement: File 1 — Experimental details and the 1H and 13C NMR spectra of 2,2’-biphen[4]arene derivatives, additional 1H NMR spectra of host–guest mixture, job plots, and the determination of the association constants. [file Beilstein_J_Org_Chem-14-2236-s001.pdf]

Supporting Information

for

Synthesis of a water-soluble 2,2′-biphen[4]arene and its efficient complexation and sensitive fluorescence enhancement towards palmatine and berberine

Xiayang Huang,[1,2] Xinghua Zhang,*[1] Tianxin Qian,[2] Junwei Ma,[2] Lei Cui[2] and Chunju Li*[1,2]

Address: [1]School of Chemical and Environmental Engineering, Shanghai Institute of Technology, 100 Hai-Quan Road, Shanghai 201418, P. R. China and [2]Department of Chemistry, Center for Supramolecular Chemistry and Catalysis, Shanghai University, Shanghai 200444, P. R. China

Email: Xinghua Zhang - xhzhang@sit.edu.cn; Chunju Li - cjli@shu.edu.cn

*Corresponding author

Experimental details and the ^{1}H and ^{13}C NMR spectra of 2,2'-biphen[4]arene derivatives, additional ^{1}H NMR spectra of host–guest mixture, job plots, and the determination of the association constants.

1. Synthesis.
2. ^{1}H NMR and ^{13}C NMR spectra of new 2,2'-biphen[4]arene compounds.
3. ^{1}H NMR spectra of **P** in the absence and presence of **2,2'-CBP4**.
4. 2D NOESY spectra of host-guest mixture of **P**/**B** with **2,2'-CBP4**.
5. Determination of the association constants.

1. Synthesis

2,2'-CBP4 was synthesized in a similar manner to a procedure of water-soluble 4,4'-biphenarene [Ref 46 in the manuscript: Org. Lett. 2016, 18, 5740–5743].

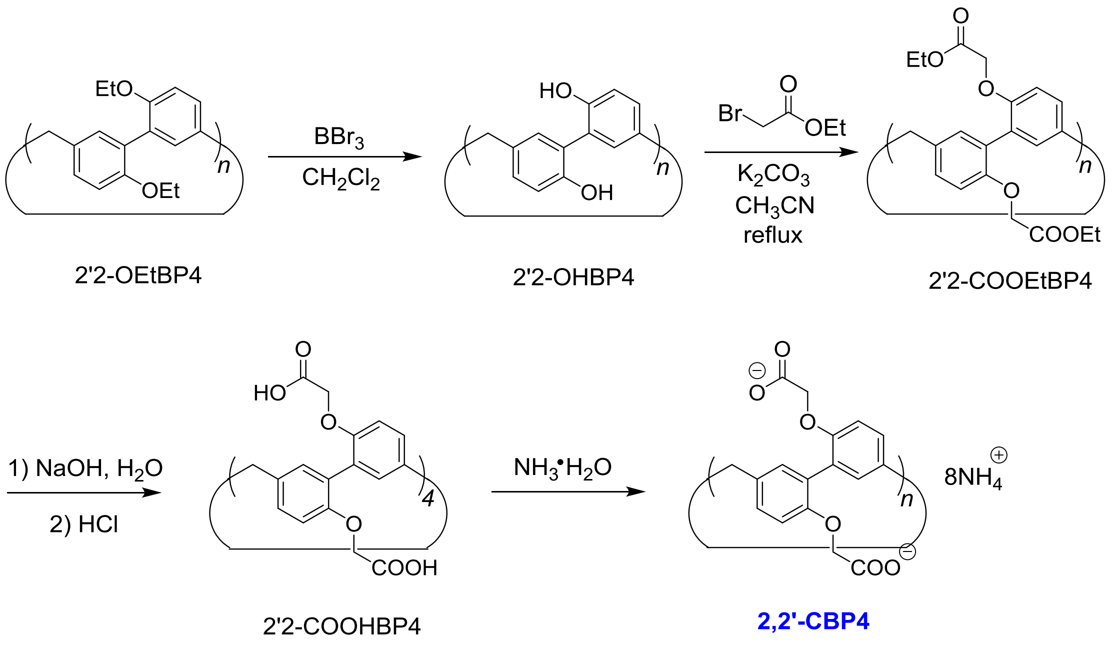

Synthetic route of **2,2'-CBP4**.

2,2'-OHBP4. To a solution of 2,2'-OEtBP4 (510 mg, 0.50 mmol) in chloroform (30 mL) was added excess boron tribromide (2.5 g, 10 mmol). The reaction mixture was stirred at room temperature for 6 h. Then the mixture was poured into ice water. The resulting precipitate was collected by filtration and washed with cold water to quantitatively obtain 2,2'-OHBP4 (390 mg). 2,2'-OHPB4. m.p. 229–230 ^oC. ^1H NMR (500 MHz, acetone-d_6): δ (ppm): 8.26 (d J = 2.16 Hz, 8H), 7.18 (d, J = 2.12 Hz, 8 H), 7.06 (dd, J = 8.25, 2.23 Hz, 8H), 6.88 (d, 8.25Hz, 8H), 3.90 (s, 8H). ^{13}C NMR (125 MHz, Acetone-d_6): δ (ppm): 152.93, 134.66, 132.92, 129.87, 127.18, 117.42, 40.73. HRMS (ESI): $C_{52}H_{40}O_8H^+$, calcd m/z 793.2796; found m/z 793.2796.

2,2'-COOEtBP4. 2,2'-OHBP4 (1.58 g, 2.0 mmol) was dissolved in CH_3CN (50 mL), and K_2CO_3 (4.10 g, 30 mmol) was added. The reaction mixture was stirred for 1 h under nitrogen atmosphere. Then ethyl bromoacetate (3.40 g, 20

mmol) was added. The mixture was heated at 80 °C for 24 hours. The reaction mixture was cooled to room temperature and filtered. The filter cake was washed with dichloromethane (60 mL × 2). The solvent was removed under vacuum. The resulting residue was dissolved in dichloromethane (60 mL), and extracted with water (30 mL × 2). The organic layer was dried using anhydrous Na_2SO_4 and concentrated. The residue was purified by recrystallization in *n*-pentane and chloroform to afford 2,2'-COOEtBP4 as a white solid (2.60 g, 88%). m.p. 183–184 °C ^{1}H NMR (500 MHz, CD_2Cl_2)：δ (ppm): 7.18 (dd, *J* = 2.23, 8.35Hz, 8H), 7.08 (d, *J* = 2.13 Hz, 8H), 6.78 (d, *J* = 8.51 Hz, 8H), 4.60 (s, 16H), 4.18 (q, *J* = 6.99, 14.28 Hz, 16H), 4.07 (s, 8H), 1.25 (t, *J* = 6.99, 13.98 Hz, 24H). ^{13}C NMR (125 MHz, CD_2Cl_2): δ (ppm): 170.42, 154.52, 135.25, 132.61, 129.25, 128.21, 112.72, 66.51, 61.28, 40.51, 15.02. HRMS (ESI): $C_{84}H_{88}O_{24}NH_4^+$, calcd m/z 1498.5982; found m/z 1498.5970.

2,2'-COOHBP4. A solution of 2,2'-COOEtBP4 (1.48 g, 1.0 mmol) in THF (20 mL) was stirred with 40% aqueous sodium hydroxide (20 mL) at 65 °C for 10 h. THF was then removed by evaporation under vacuum. The residue was diluted with deionized water (20 mL) and acidified with hydrochloric acid. The white cotton-like precipitate was collected by filtration, washed with cold water (10 mL × 3) and dried under vacuum to get 2,2'-COOHBP4 as white solid (1.09 g, 87%). m.p. > 320 °C. ^{1}H NMR (500 MHz, DMSO)：δ (ppm): 13.02 (br, 8H), 7.24 (d, *J* = 8.42Hz, 8H), 7.07 (s, 8H), 6.7 (d, *J* = 8.42 Hz, 8H), 4.69 (s, 16H), 3.97 (s, 8H). ^{13}C NMR (125 MHz, DMSO): δ (ppm): 170.58, 153.72, 133.68, 131.73, 128.33, 127.14, 112.48, 65.57. $C_{68}H_{56}O_{24}H^+$, calcd m/z 1257.3235; found m/z 1257.3237.

2,2'-CBP4. 2,2'-COOHBP4 (1.27 g, 1.0 mmol) and 20 mL of ammonium hydroxide solution (25–28%) were stirred at room temperature for 4 h. The solvent was then removed by rotary evaporation to quantitatively obtain 2,2'-CBP4 as a white solid (1.40 g, 100%). m.p. ＞320 °C. ^{1}H NMR (500 MHz, D_2O)：δ (ppm): 7.22 (s, 8H), 7.07 (s, 8H), 6.81 (s, 8H), 4.17 (s, 16H), 3.83 (s,

8H). ^{13}C NMR (125 MHz, D_2O): δ (ppm): 176.39, 153.58, 134.57, 131.73, 129.18, 127.19, 113.45, 67.87, 39.16.

2. ^{1}H NMR and ^{13}C NMR spectra of new 2,2'-biphen[4]arene compounds.

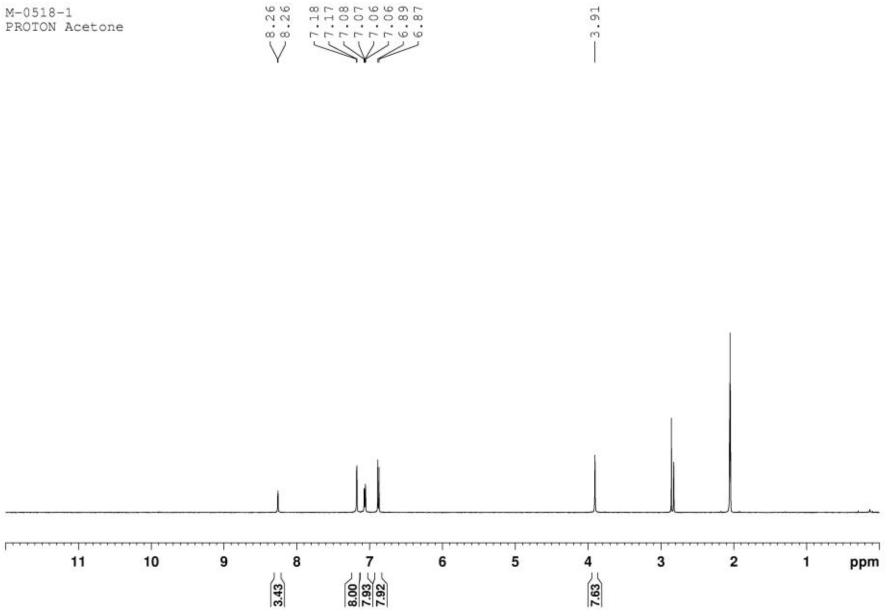

Figure S1 ^{1}H NMR spectrum (500 MHz) of 2,2'-OHBP4 in $(CD_3)_2CO$.

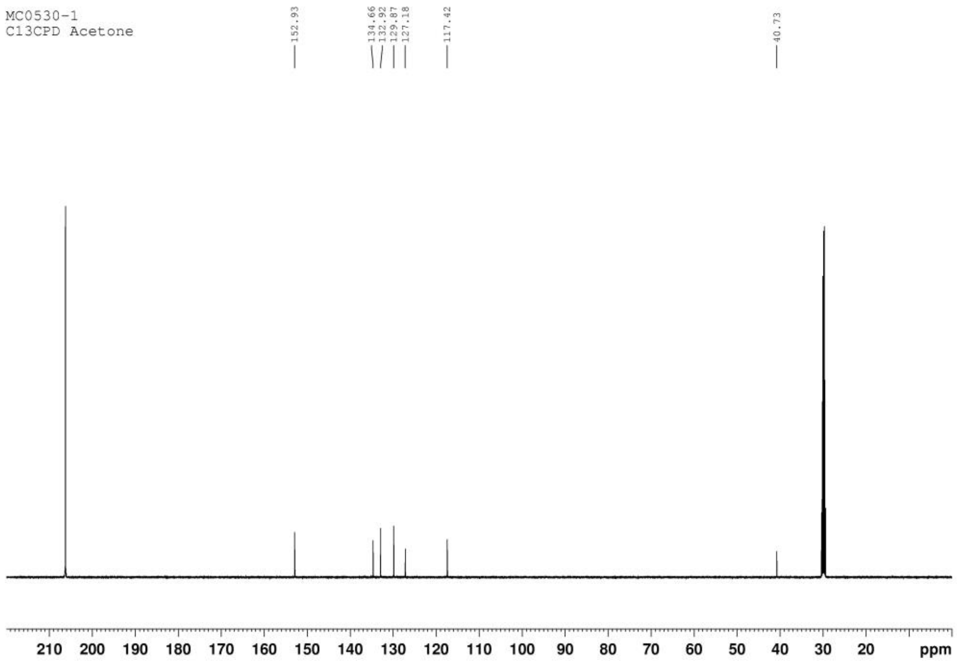

Figure S2 ^{13}C NMR spectrum (125 MHz) of 2,2'-OHBP4 in $(CD_3)_2CO$.

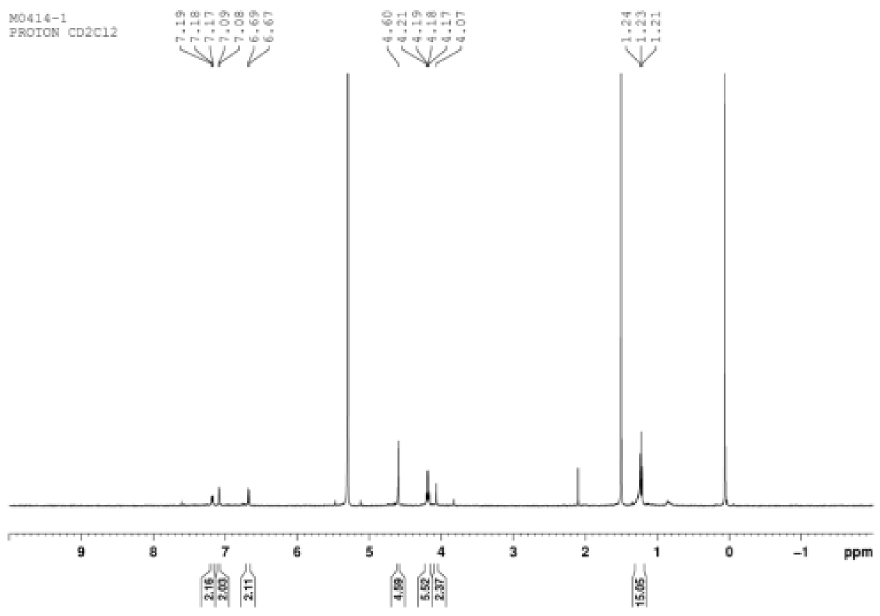

Figure S3 ^{1}H NMR spectrum (500 MHz) of 2,2'-COOEtBP4 in CD_2Cl_2.

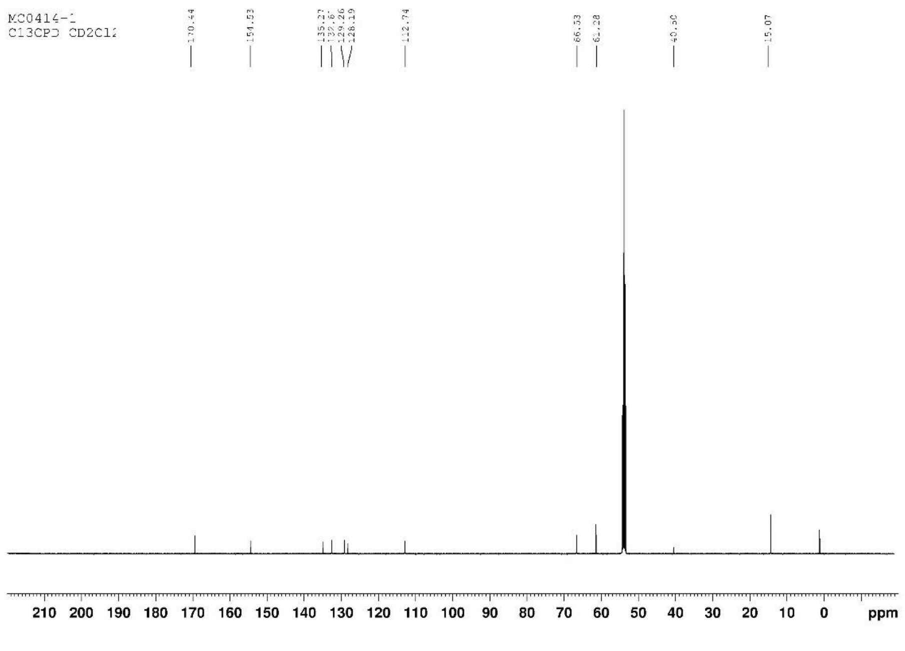

Figure S4 ^{13}C NMR spectrum (125 MHz) of 2,2'-COOEtBP4 in CD_2Cl_2.

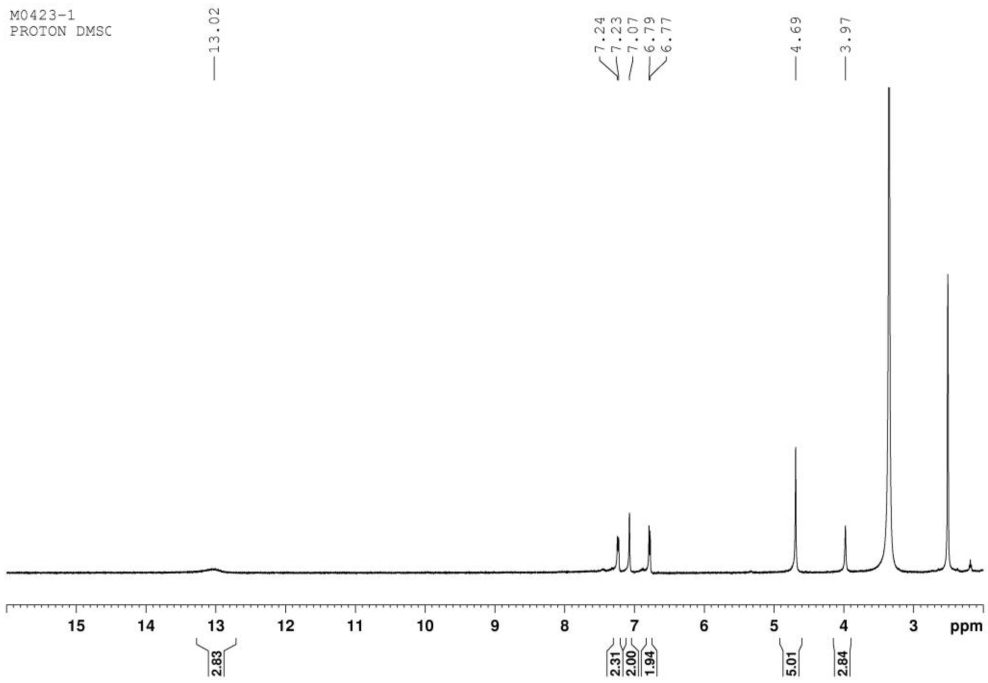

Figure S5 ^{1}H NMR spectrum (500 MHz) of 2,2'-COOHBP4 in DMSO-d_6.

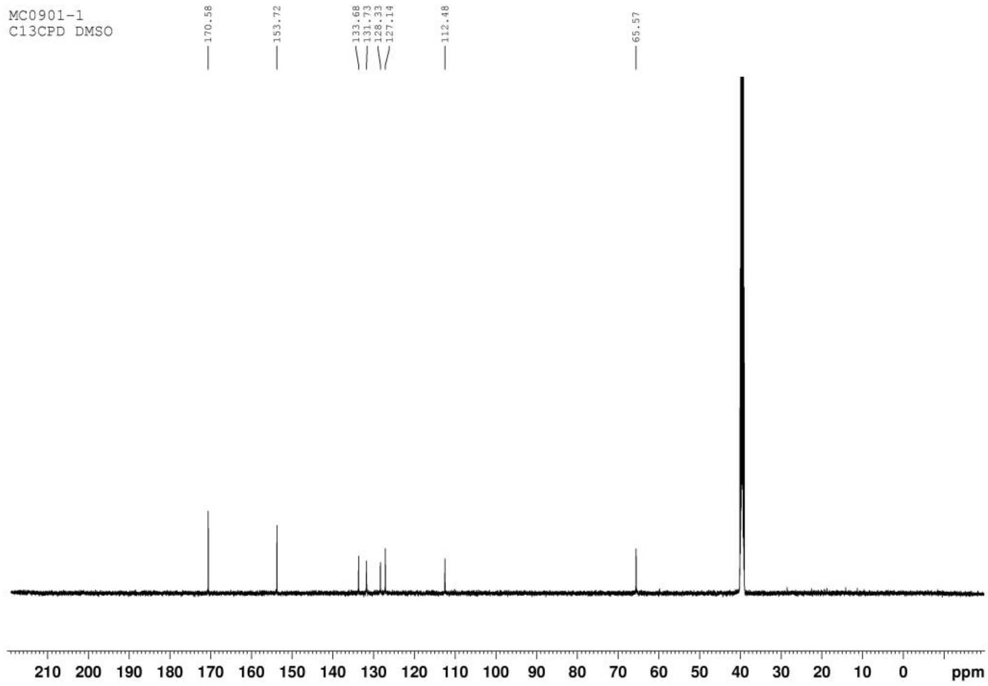

Figure S6 ^{13}C NMR spectrum (125 MHz) of 2,2'-COOHBP4 in DMSO-d_6.

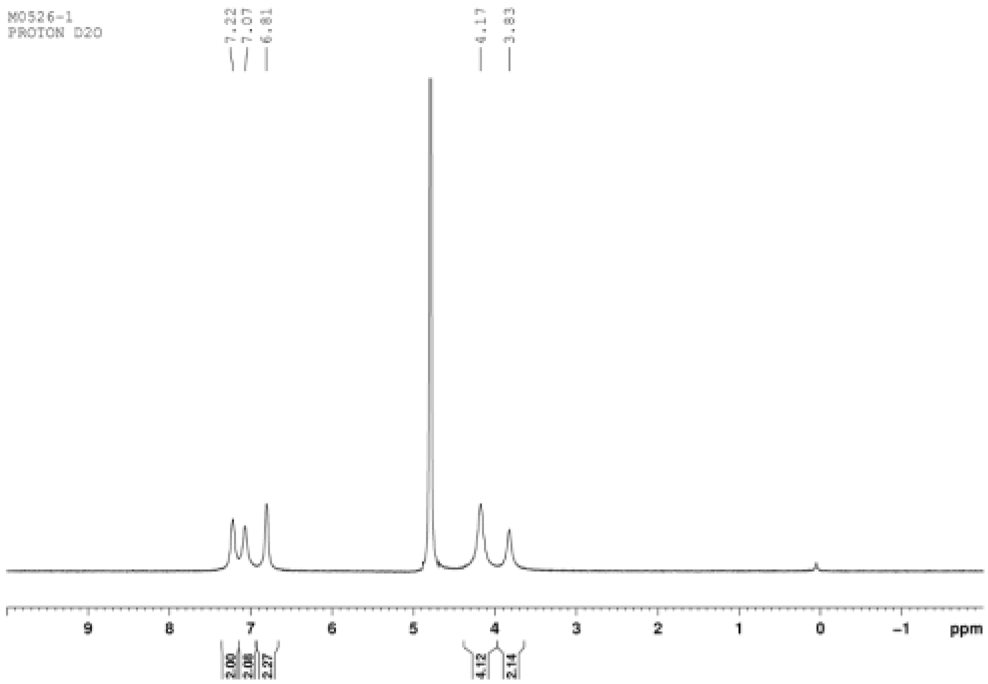

Figure S7 ^{1}H NMR spectrum (500 MHz) of **2,2'-CBP4** in D_2O.

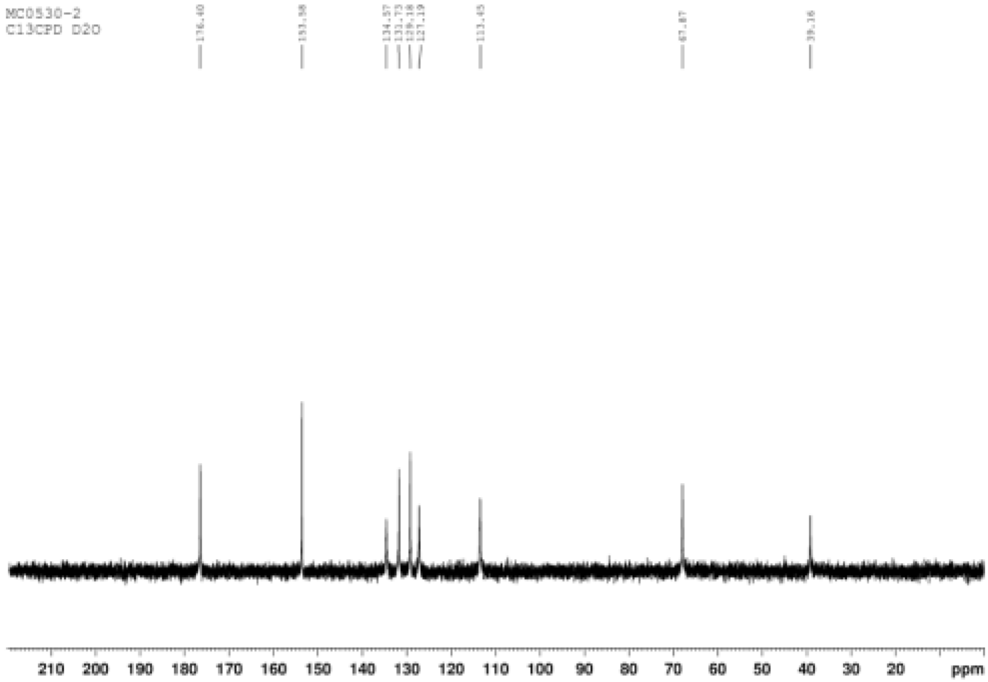

Figure S8 ^{13}C NMR spectrum (125 MHz) of **2,2'-CBP4** in D_2O.

3. ^{1}H NMR spectra of P in the absence and presence of 2,2'-CBP4.

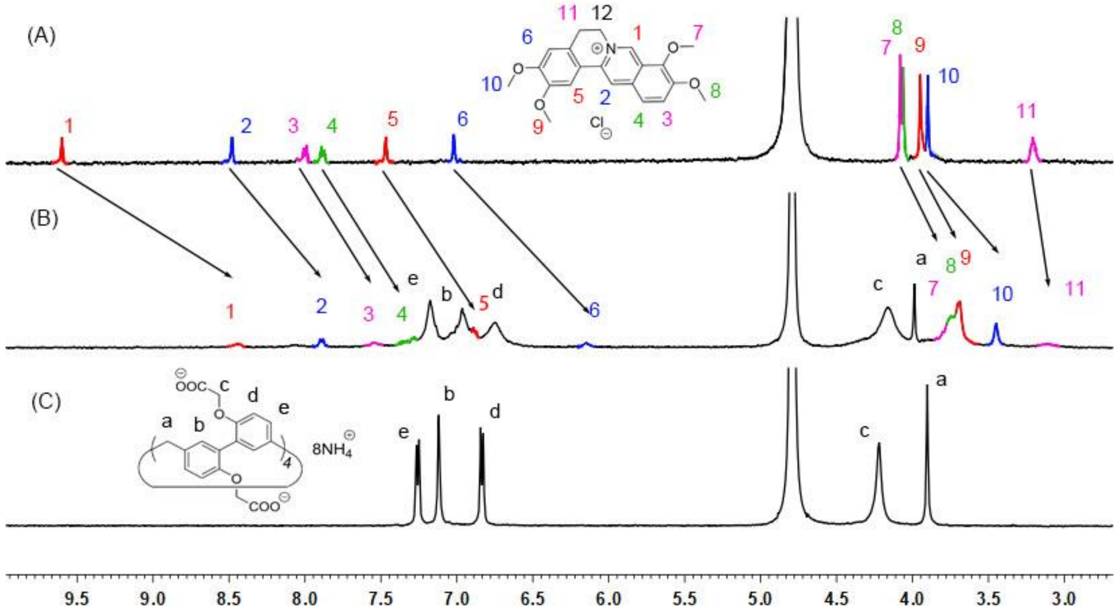

Figure S9 ^{1}H NMR spectra (500 MHz, 293 K) of (A) **P** (2.0 mM), (B) **P** (2.0 mM) + 2,2'-CBP4 (2.0 mM) and (C) 2,2'-CBP4 (2.0 mM) in deuterated phosphate buffer (pD = 7.4).

4. 2D NOESY spectra of host-guest mixture of P/B with 2,2'-CBP4.

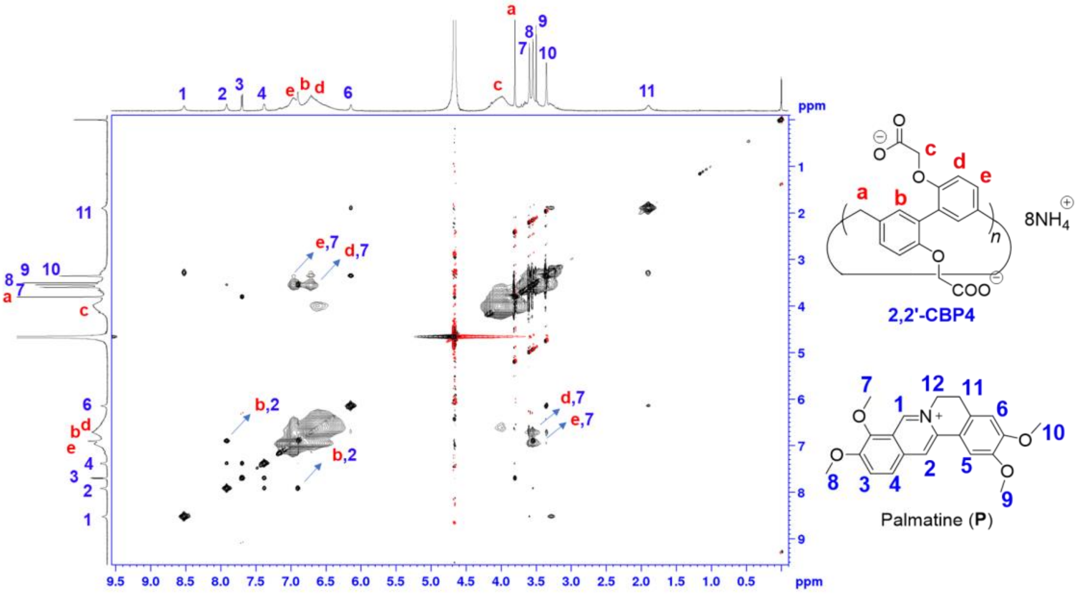

Figure S10 2D NOESY analysis (600 MHz, 293 K) of **P** (7.5 mM) with 2,2'-CBP4 (5.0 mM) in deuterated phosphate buffer (pD = 7.4) with a mixing time of 600 ms.

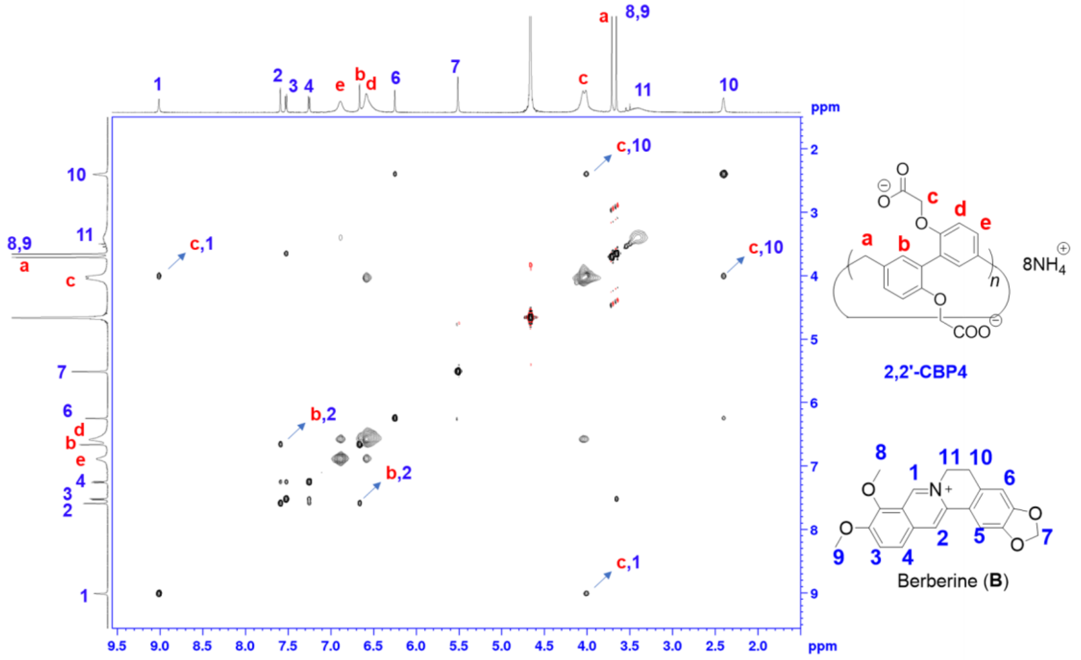

Figure S11 2D NOESY analysis (600 MHz, 293 K) of **B** (7.5 mM) with 2,2'-CBP4 (5.0 mM) in deuterated phosphate buffer (pD = 7.4) with a mixing time of 600 ms.

5. Determination of the association constants

The present of 2,2’-CBP4 could enhance **P** or **B**’s fluorescence, so the association constants (K_a) could be calculated by analyzing the fluorescence emission changes of the guest that occurred with changes in host concentration. Using the nonlinear curve-fitting method, the association constant was obtained for host–guest combination from the following equation:

$$I=I_0-(0.5((G_0/2+[host]+(1/K_a))-(sqrt((G_0/2+[host]+(1/K_a))(G_0/2+[host]+(1/K_a))-4G_0/2X))))$$

Where I is the fluorescence intensity of the system, I_0 is the intensity of the guest in the absence of 2,2’-CBP4, [host] is the initial concentration of 2,2’-CBP4.

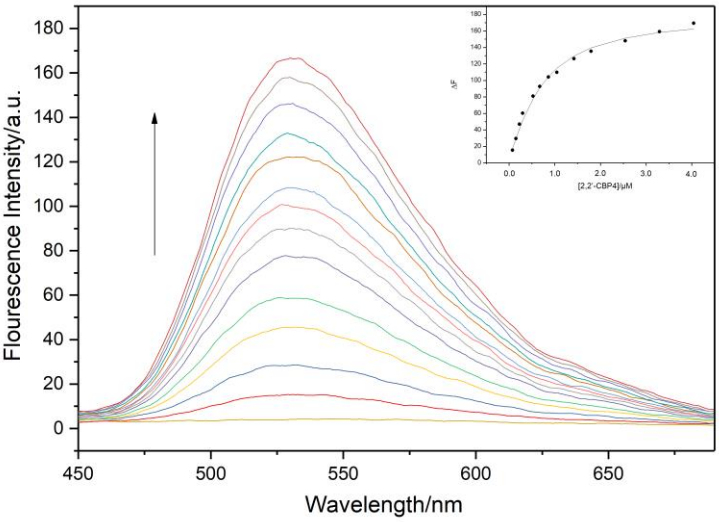

Figure S12 Fluorescence spectra of **B** in the absence and presence of 2,2’-CBP4 in aqueous phosphate buffer solution at pH 7.4 at 298 K. The excitation wavelength is at 352.0 nm. Inset: the nonlinear least-squares analysis to calculate the association constant.

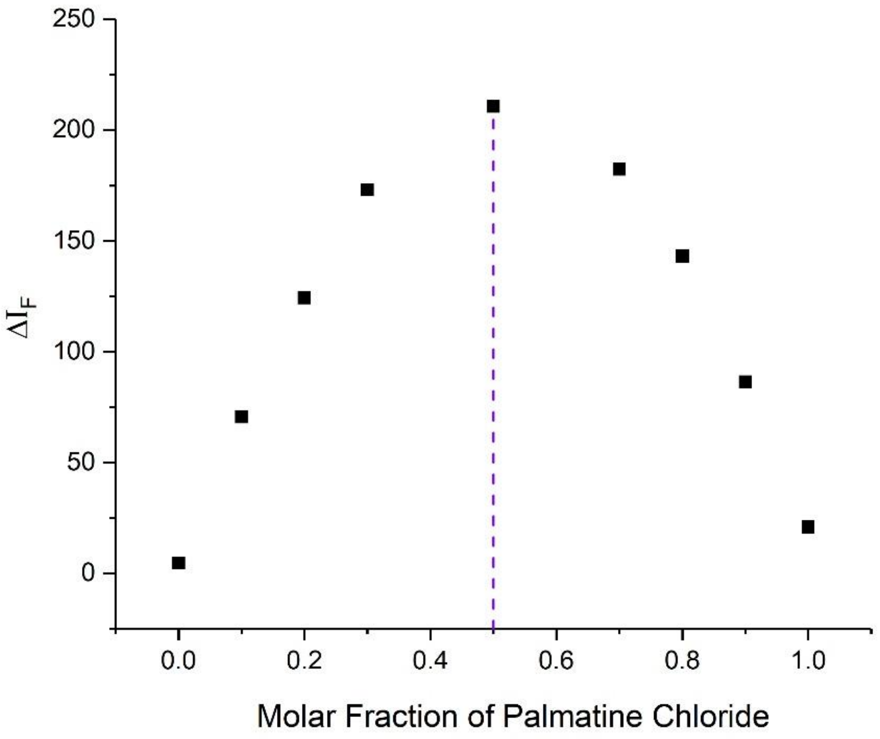

Figure S13 Job plot showing the 1:1 stoichiometry of the complex between **P** and 2,2'-CBP4 in pH 7.4 buffer by plotting the ΔI_F values against the mole fraction of **P** ([**P**]+[2,2'-CBP4]= 1.0×10^{-5} M) at 298 K.

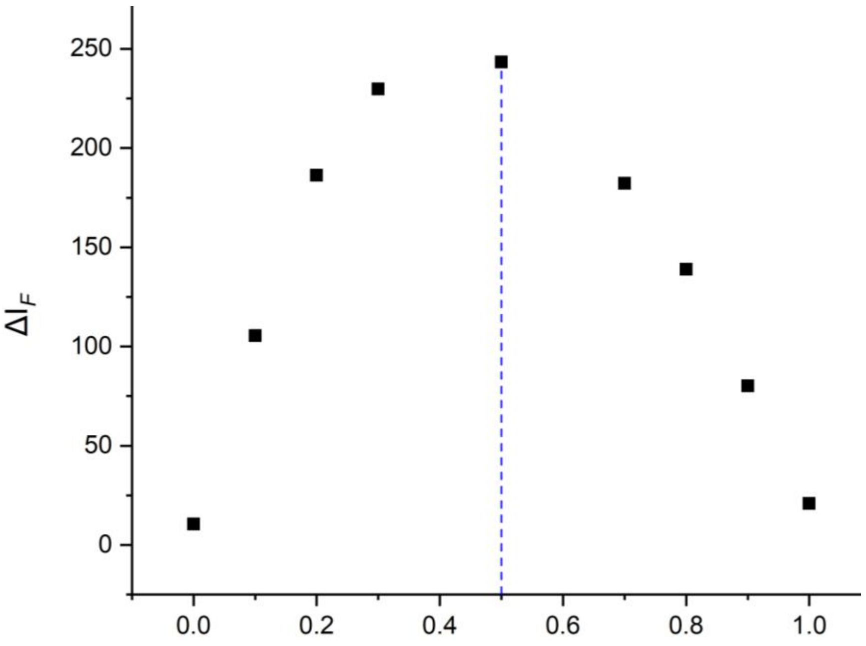

Figure S14 Job plot showing the 1:1 stoichiometry of the complex between **B** and 2,2’-CBP4 in pH 7.4 buffer by plotting the ΔI_F values against the mole fraction of **B** ([**B**]+[2,2’-CBP4]= 1.0 × 10^{-5} M) at 298 K.
